# Endodontic management of type II dens invaginatus with open apex and large periradicular lesion using the XP-endo Finisher: A case report

**DOI:** 10.4317/jced.55031

**Published:** 2018-10-01

**Authors:** Evaldo-Almeida Rodrigues, Felipe-Gonçalves Belladonna, Gustavo De-Deus, Emmanuel-João-Nogueira-Leal Silva

**Affiliations:** 1 Department of Endodontics, Fluminense Federal University, Niterói, Rio de Janeiro, Brazil. Feira de Santana State University, Feira de Santana, Bahia, Brazil; 2 Department of Endodontics, Grande Rio University, Duque de Caxias, Rio de Janeiro, Brazil; 3 Department of Endodontics, Rio de Janeiro State University, Rio de Janeiro, Rio de Janeiro, Brazil

## Abstract

Dens invaginatus (DI) represents an endodontic challenge because of its complex root canal morphology. This case report presents the clinical management of a 22-year-old woman with type II DI in right maxillary lateral incisor with a painful swelling. Pulp testing revealed no response with the tooth. Type II DI with open apex and large periradicular lesion was seen on radiograph. The treatment was planned by using cone-beam computed tomography (CBCT) imaging. Canal treatment was completed in two appointments with the aid of a dental operating microscope. In the first appointment, the internal anatomy was modified using an ultrasonic tip, and chemo-mechanical preparation was performed using the XP-endo Finisher instrument and NaOCl; calcium hydroxide intracanal dressing was used for one month. In the second appointment, an apical plug of mineral trioxide aggregate (MTA) Repair HP was performed and the remaining pulp space was then filled with gutta-percha and AH Plus sealer using the continuous wave of condensation technique. At the fourteen-month reevaluation, the patient was asymptomatic, the tooth had remained functional, and radiographic and CBCT assessment showed significant osseous healing of the lesion. Successful non-surgical management of the present type II DI was achieved in the present case. The association of CBCT, dental operating microscope, XP-endo Finisher, NaOCl and MTA Repair HP were important for ensuring a predictable outcome.

** Key words:**Cone beam computed tomography, dens invaginatus, MTA apexification, XP-endo Finisher.

## Introduction

Dens invaginatus (DI) is a developmental abnormality resulting from the invagination of enamel organ into the dental papilla, which begins at the crown and sometimes extends into the root before the occurrence of calcification (1). The prevalence of permanent teeth affected by DI is variable, ranging from 0.04 to 10%, and the most commonly affected permanent tooth is the maxillary lateral incisor (2). Oehlers (3) classified DI into 3 categories according to the depth of penetration and communication with the periodontal ligament or periapical tissue: type I - invagination confined within the crown; type II - invagination invading the root as a blind sac, with possible connection to the dental pulp; and type III - invagination penetrating through the root to open in the apical region.

Different clinical approaches have been described to treat DI, including restorative procedures, nonsurgical endodontic treatment, endodontic surgery, intentional replantation or even teeth extraction (4,5). Cases of DI teeth presenting pulp necrosis and immature apex represent a problem, as instruments with great diameter are not able to touch the root canal walls compromising debridement and disinfection; therefore, biofilm can remain in untouched areas of the irregularities of the root canals (6). Moreover, when these instruments are forced against the thin and fragile walls, there is a higher risk of root fracture (5).

Recently, a new concept-finishing instrument (XP-endo Finisher [FKG Dentaire, La Chaux-de-Fonds, Switzerland]) was developed with the purpose of improving root canal cleaning. It consists in a tip size 25 and non-tapered rotary nickel-titanium instrument made of a special proprietary alloy (MaxWire; FKG Dentaire). Because of this new alloy, the instrument changes its shape according to the temperature: in room temperature, in its martensitic phase (M-phase), the instrument stands straight; however, when submitted to body temperatures, the file changes to its austenitic phase (A-phase) assuming a spoon-shape of 1.5 mm depth in the final 10 mm of its length. According to the manufacturer, the A-phase shape allows the instrument to access and clean areas that other instruments might not have reached, without damaging dentin or altering the original canal shape (7). This concept might be interesting to clean teeth with large canals and thin walls, preventing the increase of root fragility. This case report describes the clinical management of a nonsurgical endodontic treatment performed with the XP-endo Finisher instrument in a maxillary lateral incisor presenting type II DI.

## Case Report

**Figure 1:**
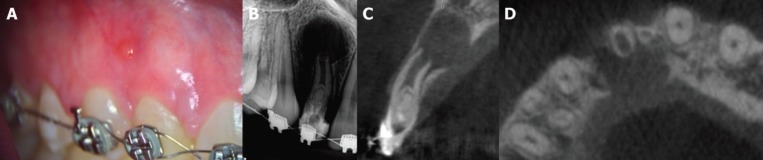
A. Clinical examination showing a sinus tract. B. Radiographic examination showing a large periradicular radiolucency, an open apex and a complex canal anatomy with type II DI. C. Sagittal and D. axial CBCT images showed the invagination extending beyond the cementoenamel junction, reaching the pulpal space and periapical radiolucency disrupting the bone cortical palatal.

A 22-year-old woman with no general health problems was referred by her dentist to the Endodontic Department, School of Dentistry, Feira de Santana State University. The patient complained of painful swelling on the mucosa over the maxillary right lateral incisor. Clinical examination revealed a sinus tract (Fig. 1A), and the tooth did not respond to cold thermal test (Endo-Frost; Roeko, Langenau, Germany), as well as presented hypersensitive response to percussion and palpation, although adjacent teeth all responded within normal patterns. Periodontal probing depths were normal (< 3 mm).

Radiographic examination revealed a large periradicular radiolucency, an open apex and a complex canal anatomy with type II DI (Fig. 1B). Cone-beam computed tomographic (CBCT) scan was requested as a complementary examination to avoid possible complications during treatment (8). Sagittal and axial CBCT images showed the invagination extending beyond the cementoenamel junction, reaching the pulpal space and periapical radiolucency disrupting the bone cortical palatal (Fig. 1C,D). The patient was informed of the diagnosis and the need for root canal treatment.

**Figure 2:**
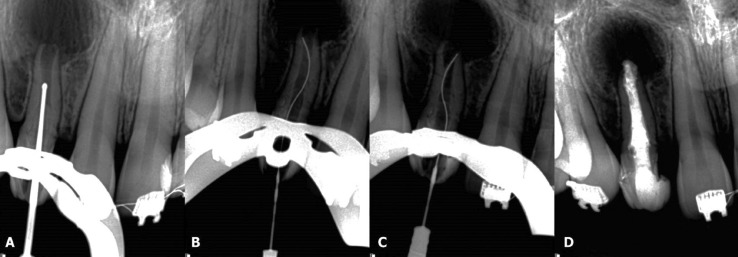
A. An ultrasonic tip was carefully used to remove the invaginated tissue. B and C. Radiographs showing the XP-endo Finisher instrument touching the canal walls. D. Final radiograph presenting the root canal filling of type II DI.

Root canal treatment was performed under local anesthesia using 2% lidocaine with 1:100,000 epinephrine (Nova DFL; Taquara, Rio de Janeiro, Brazil). A rubber dam was placed, and the access cavity was performed using diamond burs in high-speed rotation. The invaginated tissue was carefully removed using an ultrasonic tip (E3D; Helse Dental Technology, São Paulo, Brazil) (Fig. 2A) with the aid of a dental operating microscope (Alliance; São Carlos, São Paulo, Brazil). The canal was copiously irrigated with 2.5% NaOCl and the working length (WL) was established electronically with an apex locator (Root ZX; J Morita USA Inc, Irvine, CA) using a size 50 K-file (Dentsply Maillefer, Ballaigues, Switzerland). The XP-endo Finisher instrument was placed in a contra-angle hand piece (VDW, Munich, Germany), cooled (Endo-Frost), removed from the plastic tube and inserted in the canal without rotation. Then, the rotation was initialized (800 rpm and 1 Ncm), and the instrument was activated for 1 min using a slow and gentle 7-8 mm lengthwise movements up to the WL. The instrument was brushed against the sidewalls of the canals during the instrumentation (Fig. 2B,C). This cycle was repeated three times. The canal was irrigated with 5 mL of 2.5% NaOCl using a 30-G NaviTip needle (Ultradent Products Inc, South Jordan, UT, USA) up to 3 mm short of the WL after each cycle. The smear layer was removed by rinsing the canal with 2 mL of 17% EDTA for 3 min followed by 5 mL of 2.5% NaOCl. Finally, a 3 mL rinse with bidistilled water was used in the final irrigation to flush out the NaOCl. After that, the canal was dried with sterile absorbent paper points (Dentsply Maillefer), filled with calcium hydroxide (Ultracal; Ultradent Products Inc), and sealed with a temporary filling material (Cavit; 3M ESPE, Seefeld, Germany).

**Figure 3:**
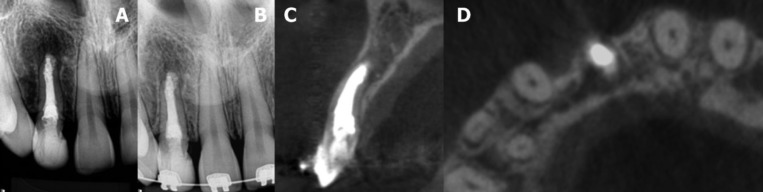
A. The patient returned for clinical and radiographic examinations after six (a) and B. fourteen months. C. Sagittal and D. axial CBCT images of a new scan performed after fourteen months, showing advanced healing and periapical repair.

One month later, at the second appointment, the tooth was asymptomatic and the sinus tract had disappeared. After access, the canal was copiously irrigated with 2.5% NaOCl and another cycle using the XP-endo Finisher instrument was performed to remove the calcium hydroxide. An apical plug of mineral trioxide aggregate (MTA) Repair HP (Angelus Dental Solutions; Londrina, Paraná, Brazil) with approximately 3 mm was performed. The remaining pulp space was then carefully filled with gutta-percha (VDW) and AH Plus sealer (Dentsply De Trey, Konstanz, Germany) using the continuous wave of condensation technique. The continuous wave of condensation technique was performed using a fine tip plugger of the System B, which was heated to 200°C and taken to a depth 6-mm short of the apical plug. The tip was allowed to cool for 15 s, and a single burst of heat was applied for 1 s and the tip was removed. The canal was completely backfilled with Obtura II gutta-percha (Spartan, Fenton, MO) with the unit set at 200°C.The access was sealed with a temporary filling material (Cavit), a final radiographic was performed (Fig. 2D) and the patient was referred to her general dentistry. The patient returned for clinical and radiographic examinations after six (Fig. 3A) and fourteen months (Fig. 3B). Another CBCT scan was performed after fourteen months (Fig. 3C,D). Periapical radiograph and CBCT images revealed evidence of advanced healing and periapical repair.

## Discussion

The major goal of root canal therapy is to remove microorganisms from the root canals to prevent or heal apical periodontitis (6). This is currently done by mechanically shaping and chemically cleaning the root canal system. When wide root canals are present, the preparation procedure can fail to adequately clean and shape these canals, because the instrument does not touch most of the walls, and thus it is difficult to remove the biofilm (9). In this case report, besides the inherent difficulties for a root canal treatment of DI due to its complex internal anatomy, a large canal with open apex and showing a large periradicular lesion was present.

The introduction of CBCT in Endodontics has contributed greatly to the planning, diagnosis, therapy, and prognosis of cases where there are complex conditions such as the presence of anatomical anomalies (10). In these cases, the use of CBCT is very helpful to recognize the type of DI, to reveal size and depth of the invagination as well as to determine the dimensions of bone lesion and to follow up the healing. According to the Position Statement of American Association of Endodontists and American Academy of Oral and Maxillofacial Radiology on the use of CBCT in Endodontics (8), limited field of vision (FOV) CBCT should be considered the imaging modality of choice for initial treatment of teeth with suspected complex morphology and dental anomalies; in addition, in the absence of signs and symptoms, if limited FOV CBCT was the imaging modality of choice at the time of evaluation and treatment, it may be the imaging modality of choice for follow-up evaluation. Therefore, both preoperative and postoperative CBCT scans were performed herein.

In the present case report, the XP-endo Finisher instrument was used during chemo-mechanical preparation. According to the manufacturer, due to its special proprietary alloy (MaxWire), the XP-endo Finisher instrument assumes a spoon-shape at body temperature, performing debridement on hard-to-reach areas of the canal system. This specific feature of the instrument could be visualized in trans-operative radiographs (Fig. 2B,C). In addition, this instrument does not remove tooth structure and, consequently, it does not damage dentin or alter the original canal shape (7,9). Moreover, recent studies highlighted its disinfection abilities, in which the XP-endo Finisher instrument showed a better efficacy on biofilm removal than conventional needle irrigation and passive ultrasonic irrigation (9) and it also promoted significant reduction in bacterial counts after chemomechanical preparation when compared to passive ultrasonic irrigation (11). That is why the XP-endo Finisher instrument was used to shape and clean the present case of type II DI.

Endodontic management of a permanent tooth with open apex, pulp necrosis, and periapical lesion consists on apexification or revascularization. Some studies have compared these procedures and have shown that revascularization was not superior to other apexification techniques in either clinical or radiographic outcomes (12). MTA apexification and revascularization provides a reliable outcome in the aspects of resolution of the disease and tooth functional retention, but none of these treatments provide satisfactory predictable further root development (13). A recent systematic review with meta-analysis compared endodontic treatments available in the management of immature necrotic permanent teeth; the authors conclude that MTA apexification seems to produce overall better clinical and radiographic success rates (14). In this case report, a one-step apexification with MTA was performed. This approach is in line with previous studies that reported a high rate of success in both single or multiple-visits appointment treatments for teeth with open apex when an apical MTA plug was performed (15). MTA Repair HP was used herein due to its better handling when compared to White MTA. One of the differences of this new material over its predecessor was the replacement of the distilled water by a liquid containing water and an organic plasticizer. This difference in the formulation provides a higher plasticity, improving handling and insertion of the material.

In conclusion, a successful non-surgical management of type II DI with wide canal, open apex, and large periradicular lesion was achieved. The association of CBCT, dental operating microscope, XP-endo Finisher, NaOCl and MTA Repair HP were important for ensuring a predictable outcome with a successful 14 months’ follow-up showing periapical healing.
